# Mitochondria and α-Synuclein: Friends or Foes in the Pathogenesis of Parkinson’s Disease?

**DOI:** 10.3390/genes8120377

**Published:** 2017-12-08

**Authors:** Gaia Faustini, Federica Bono, Alessandra Valerio, Marina Pizzi, PierFranco Spano, Arianna Bellucci

**Affiliations:** 1 Department of Molecular and Translational Medicine, University of Brescia, 25123 Brescia, Italy; g.faustini004@unibs.it (G.F.); alessandra.valerio@unibs.it (A.V.); marina.pizzi@unibs.it (M.P.); pierfranco.spano@unibs.it (P.S.); 2 Laboratory of Personalized and Preventive Medicine, University of Brescia, 25123 Brescia, Italy; federica.bono@unibs.it

**Keywords:** Parkinson’s disease, mitochondrial dysfunction, mitochondrial homeostasis, α-synuclein, dopaminergic neurons

## Abstract

Parkinson’s disease (PD) is a movement disorder characterized by dopaminergic nigrostriatal neuron degeneration and the formation of Lewy bodies (LB), pathological inclusions containing fibrils that are mainly composed of α-synuclein. Dopaminergic neurons, for their intrinsic characteristics, have a high energy demand that relies on the efficiency of the mitochondria respiratory chain. Dysregulations of mitochondria, deriving from alterations of complex I protein or oxidative DNA damage, change the trafficking, size and morphology of these organelles. Of note, these mitochondrial bioenergetics defects have been related to PD. A series of experimental evidence supports that α-synuclein physiological action is relevant for mitochondrial homeostasis, while its pathological aggregation can negatively impinge on mitochondrial function. It thus appears that imbalances in the equilibrium between the reciprocal modulatory action of mitochondria and α-synuclein can contribute to PD onset by inducing neuronal impairment. This review will try to highlight the role of physiological and pathological α-synuclein in the modulation of mitochondrial functions.

## 1. Introduction

Parkinson’s disease (PD), the most common neurodegenerative movement disorder, is characterized by a variety of premotor signs, as well as typical motor symptoms, such as resting tremor, rigidity, bradykinesia and postural reflex impairment. A key neuropathological hallmark of PD is the progressive loss of nigrostriatal dopaminergic neurons, which is thought to begin from synaptic terminal degeneration [1]. In addition, the brain of affected patients exhibits the presence of proteinaceous inclusions, named Lewy bodies (LB) and Lewy neuritis (LN), mainly composed of fibrillary aggregated α-synuclein [2]. To date, pathological inclusions containing α-synuclein, either in the form of LB and LN or others, are typical of a series of neurodegenerative disorders, including dementia with LB (DLB), multiple system atrophy (MSA), LB dysphagia, Alzheimer's disease (AD) and its LB variant (LBVAD), as well as neurodegeneration with brain iron accumulation type-1 (NBIA-1) [3,4,5]. These are commonly referred as synucleinopathies.

For many years, the severity and the duration of motor symptoms in PD has been thought to directly correlate with the rate of neuronal cell loss in the substantia nigra pars compacta (SNpc) [6]. Nonetheless, recent compelling evidence supports that synaptic and axonal degeneration might be the crucial early pathological events underlying symptom onset and initiating nigrostriatal neuron loss in PD [7,8]. In this context, the massive amount of insoluble α-synuclein observed at striatal dopaminergic terminals, which is orders of magnitude higher than those detected in LB [9], hints that synapses may even be the primary site of accumulation of α-synuclein. These findings support that the deposition of insoluble α-synuclein at the presynaptic endings of nigrostriatal neurons may constitute the *primum movens* for the retrograde pattern of degeneration. 

This notwithstanding, the mechanisms through which α-synuclein accumulation drives dopaminergic neuronal cell death are still enigmatic. Among the possible mechanisms involved in neurodegeneration in PD, there are alterations of protein degradation systems, synaptic failure and collapse and mitochondrial dysfunction [10,11]. 

Nigrostriatal neurons show large axonal lengths with an enormous number of synapses [12,13]. For this reason, they require a gargantuan energy demand as they also need to bolster their intrinsically elevated electrical excitability [13]. This hypothesis is sustained by evidence showing that mitochondrial oxidative stress is higher in the axons of nigrostriatal dopaminergic neurons rather than in those of noradrenergic neurons of the ventral tegmental area. Consistently, the reduction of nigrostriatal neuronal arborization decreases the phenomenon of mitochondrial oxidative phosphorylation and neuron vulnerability [14]. However, multiple factors, such as broad spikes, pacemaking, low intrinsic Ca^2+^ buffering, cytosolic Ca^2+^ oscillations and dopamine oxidation, need to occur simultaneously in order to render dopaminergic neurons vulnerable to α-synuclein/mitochondrial dysfunction-linked neurodegeneration [13,15]. Therefore, the dichotomy between the exact contribution of α-synuclein deposition and mitochondria dysfunction to dopaminergic neurons degeneration still constitutes an issue to be solved.

## 2. Parkinson Disease α-Synuclein’s Pathology and Its Relation with Neuronal Degeneration

Numerous studies indicate α-synuclein as the major agent in PD pathophysiology [16,17]. In particular, α-synuclein aggregation may be the main cause of synaptic dysfunction and of the associated neurodegeneration [9,18]. A large amount of α-synuclein within LB is truncated at the *C*-terminal domain [19,20,21]. Of note, truncated α-synuclein has a higher propensity to aggregate when compared to either wild-type (wt) or mutated forms of the protein [22]. Moreover, even modifications of *C*-terminal domain may increase the propensity of the protein to aggregate [23,24]. The centrality of α-synuclein in the pathogenesis of PD is further supported by the fact that patients showing multiplication or missense mutations in α-synuclein gene (*SNCA*), such as E46K, A30P and A53T [25], develop early onset forms of PD characterized by peculiar clinical and histopathological features [26,27].

However, 90% of PD cases manifest with sporadic onset, thus implying that multiple factors contribute to α-synuclein accumulation in this disorder. Emerging data from human and animal models of PD highlight a role for α-synuclein in the control of neuronal mitochondrial dynamics [28,29]. The effect of overexpression of α-synuclein on mitochondrial functions has been investigated in human wt, mutated or truncated α-synuclein transgenic mice, even when generated by the use of viral vectors, where α-synuclein accumulation has been found to result in different PD-like phenotypes [30,31,32].

However, even if brain α-synuclein deposition is thought to constitute a central event in the pathogenesis of PD, α-synuclein pathology is not confined within the nigrostriatal system in the brain of affected subjects. LB or LB-like aggregates composed of α-synuclein have been found to accumulate in the peripheral nervous system and in the dorsal root ganglia of PD patients [33,34,35]. Moreover, α-synuclein accumulation within the peripheral nervous system can also occur in neurologically intact aged individuals [36]. For this reason, PD and aging have been proposed to be a unique entity, with patients manifesting PD when pathological alterations and neurodegeneration overwhelm a critical threshold. Indeed, neuropathological accumulation of LB has been detected in the post-mortem brains of non-parkinsonian aged subjects, so that it has been suggested that, if they had lived long enough, they would have developed PD or other forms of parkinsonism [37]. This idea fits with the recently introduced threshold theory of PD, according to which α-synuclein deposition presents a widespread distribution, and may increase predisposition to the onset of prodromal premotor or motor symptoms by inducing the impairment or loss of distinct populations of peripheral or central neurons [38]. In particular, PD preclinical and clinical symptoms would occur only when the distinct functional reserves of neurons became no longer able to support their actions. In this context, the onset of motor symptoms originating from α-synuclein accumulation at nigrostriatal neurons would be delayed, when compared to that the prodromal signs related to α-synuclein increase in peripheral neurons. This can be ascribed to a larger functional reserve of SNpc neurons coupled to that of basal ganglia circuits. However, the above-cited dopaminergic cell-autonomous risk factors can contribute to the selective loss of brain nigrostriatal neurons [39].

## 3. Mitochondria Alterations in Parkinson’s Disease

PD prevalence clearly increases along with aging, therefore the age-related progressive accumulation of molecular damage, which is associated with an impairment of proteasome activity, autophagy and mitochondrial dysfunction [40,41], is thought to be relevant during disease onset. Mitochondrial deficits have largely been described as crucial pathogenic events in the pathogenesis of PD [28,29]. The first evidence supporting the involvement of mitochondria dysfunction in PD pathogenesis was complex I deficiency and impaired activity in the SNpc of patients [42,43]. Later on, complex I deficiency was found to be associated with the oxidation of complex I subunits and the consequent increase of reactive oxygen species (ROS) production and oxidative damage [44]. Other authors reported reduced complex I activity in the skeletal muscle [45,46] and platelets of PD patients [47,48], supporting the hypothesis that systemic mitochondria alterations are implicated in PD. Along the same lines, cultured fibroblasts isolated from PD patients display impaired oxidative decarboxylation of pyruvate [49]. Complex I is composed by a large number of mitochondrial and nuclear DNA-encoded subunits that are damaged in PD patients [50]. Interestingly, polymorphisms in mitochondrial DNA-encoded complex I have been found to constitute susceptibility factors for PD [51], and mitochondrial DNA mutations can cause parkinsonism-like alterations in mitochondrial DNA polymerase γ [52].

Neurotoxins inhibiting mitochondrial complex I, such as 1-methyl-4-phenyl-1,2,3,6-tetrahydropyridine (MPTP) and rotenone, have been used to induce oxidative damage and parkinsonian-like symptoms in various animal models of PD [31]. Moreover, the lack of the NADH:ubiquinone oxidoreductase iron-sulfur protein 4 (Ndufs4) subunit of complex I decreases dopamine release in animal models and induces a higher vulnerability to MPTP [53]. 

Finally, mutations in genes encoding mitochondrial-related proteins such as DJ-1 and PINK-1 have been associated with the onset of familial forms of PD [54].

## 4. Mitochondria and α-Synuclein: Reciprocal Modulation

Recent evidence supports that that the interaction between α-synuclein and complex I reduces mitochondrial activity, while accumulation of α-synuclein into mitochondria can lead to their dysfunction and α-synuclein overexpression has been associated with complex I dysfunction [55,56]. The *N*-terminal domain of α-synuclein has been found to be relevant to target the protein to complex I [55] and domain integrity is necessary for the control of neuronal mitochondrial morphology [57,58].

Recently, it has been reported that a fraction of soluble α-synuclein is targeted to mitochondria and appears to interact directly with mitochondria-associated endoplasmic reticulum membranes (MAM) [59]. The fraction of α-synuclein that directly interacts with mitochondria can influence mitochondrial fusion and fission, and overexpression of the protein affects mitochondrial morphology by producing mitochondrial fragmentation in cell-based models of PD [60]. 

The interaction of α-synuclein oligomers with mitochondrial membranes is followed by a decline in mitochondrial respiration and neuronal cell death [60]. Overexpression or mutations of α-synuclein compromise mitochondrial function by acting on several mechanisms, including increased mitophagy [28,56]. Furthermore, overexpression of α-synuclein in mouse models induces the formation of toxic aggregates that, by directly inhibiting translocase of the outer membrane 20 (TOM20), impair mitochondrial protein import [61]. Notably, α-synuclein/TOM20 interaction and impaired mitochondrial protein import have been also detected in the SNpc neurons of PD patients [61]. Overexpression of α-synuclein also causes release of cytochrome c from mitochondria, generating a calcium and nitric oxide increase with consequent oxidative modifications of mitochondrial components [62]. Along the same lines, α-synuclein fibrils have been found to impair mitochondrial function through upregulation of inducible nitric oxide synthase (iNOS) and nitric oxide generation, which contribute to degeneration by increasing protein nitration levels [63]. In addition, α-synuclein prevents the inhibition of pro-apoptotic pathways through modulation of mitogen-activated protein kinase (MAPK) [64].

Epidemiologic studies have shown association between environmental factors (e.g., exposure to pesticides such as paraquat and rotenone) and PD onset [65]. This has been confirmed by experimental studies that have demonstrated that exposure of mice or rats to these toxins can result in PD-like phenotypes encompassing α-synuclein deposition and aggregation [66,67,68]. Notably, further evidence has confirmed that the direct interaction of these agrochemicals with α-synuclein does not increase the fibrillation of the protein, thus fostering the idea that the effect of these compounds in PD is related to the inhibition of mitochondrial complex I and/or the up-regulation of α-synuclein [69].

Similarly, the effect of MPTP on mitochondria deficiency is mediated by α-synuclein overexpression and aggregation. Indeed, the presence of elevated levels of α-synuclein fosters the inhibitory action of MPTP or rotenone on mitochondrial complex I [29] and human mutated α-synuclein transgenic mice display increased sensitivity to MPTP administration [70]. Contrariwise, α-synuclein gene knockout or silencing prevents the dopaminergic neuron loss mediated by MPTP induction [71]. However, rotenone has been found to bypass α-synuclein knockout resistance. Indeed, α-synuclein deficient mice are more sensitive to the neurotoxic action on complex I inhibition by rotenone, and show a more marked degeneration of dopamine neurons when compared to wt mice [72]. Other authors have reported that α-synuclein silencing in dopaminergic neurons confers resistance to MPTP with the increased cell survival resulting from alteration of cellular dopamine homeostasis by reduction of dopamine uptake [73]. These findings support a synaptic-specific effect of α-synuclein-mediated mitochondria control.

Mice knockout for α-synuclein has also been found to show decreased complex I/III activity resulting from α-synuclein-mediated alterations of mitochondrial membrane lipid composition [74]. An impaired connectivity between complex I and III and α-synuclein has been confirmed, even in human fetal dopaminergic primary neuronal cells exposed to α-synuclein gene silencing, thus supporting a role of α-synuclein in physiological mitochondria respiration [55]. Finally, α-synuclein physiologically interacts and acts on adenosine triphosphate (ATP) synthase [75]. This interaction may of course be also relevant for the control of mitochondrial homeostasis.

Collectively, these observations support that α-synuclein can physiologically control mitochondrial function by regulating mitochondrial complexes. For this reason, alterations of the protein can contribute to bioenergetic defects that coincide with mitochondrial dysfunctions and PD onset. 

## 5. Conclusions

Mitochondrial alterations and α-synuclein pathological deposition play a central role in PD. Numerous studies have shed light upon the fact that a critical interplay exists between mitochondria and α-synuclein in both physiological and pathological condition. α-Synuclein is emerging as a key physiological modulator of mitochondrial homeostasis. 

**Figure 1:**
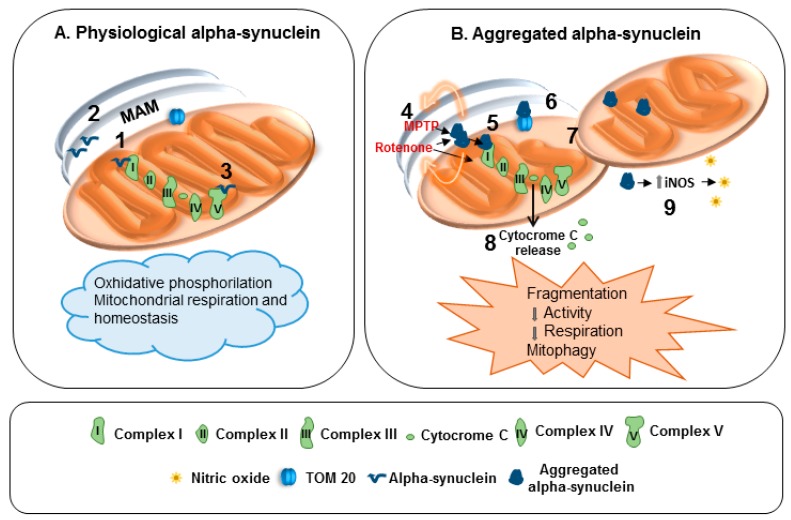
Effects of physiological and aggregated α-synuclein on mitochondria. (**A**) **1:** α-Synuclein controls protein targeting and mitochondrial morphology. **2:** Interaction with mitochondria-associated ER membranes (MAM). **3:** Interaction with adenosine triphosphate (ATP) synthase. (**B**) **4:** Inhibitory action of 1-methyl-4-phenyl-1,2,3,6-tetrahydropyridine (MPTP) and Rotenone on complex I is fostered by α-synuclein. **5:** Complex I dysfunction. **6:** Impaired mitochondrial protein import. **7:** Mitochondrial fragmentation and mitophagy. **8:** Release of cytochrome C. **9:** Upregulation of inducible nitric oxide synthase (iNOS) and nitric oxide generation.

Indeed, α-synuclein physiologically interacts with MAM and controls mitochondrial functions and morphology, even by interacting with complex I and ATP synthase (Figure 1A). When α-synuclein forms aggregates the functions of complex I are compromised (Figure 1B). Furthermore, α-synuclein aggregation and loss of function can crucially impair mitochondria homeostasis. In particular, they reduce mitochondrial protein import, and prompt mitochondria fragmentation, mitophagy, release of cytochrome c as well as nitrosative stress via iNOS induction (Figure 1B).

This notwithstanding, mitochondrial dysfunctions may lead to α-synuclein aggregation and deposition. Therefore, mitochondrial health is relevant to ensuring the proper fulfillment of α-synuclein physiological actions. Mitochondria and α-synuclein are thus revealed as good friends, both contributing to ensuring the correct functions of healthy neurons. However, imbalances in α-synuclein/mitochondria reciprocal-modulatory system can easily lead to neuronal impairment. In this scenario, mitochondrial alterations and α-synuclein aggregation can both compromise neuronal cell resilience through the establishment of a vicious self-propagating circle, where one can foster the detrimental action of the other in an unfinished fight. The study of α-synuclein/mitochondria interplay in health and disease is thus pivotal for our understanding of their biological functions, and hopefully for the identification of novel therapeutic targets for PD.
